# Efficacy of Ethanolic Extract of *Syzygium aromaticum* in the Treatment of Multidrug-Resistant *Pseudomonas aeruginosa* Clinical Isolates Associated with Urinary Tract Infections

**DOI:** 10.1155/2021/6612058

**Published:** 2021-06-15

**Authors:** Osama Ahmed, Hassan Mohamed, Wesam Salem, Magdy Afifi, Yuanda Song

**Affiliations:** ^1^Department of Botany and Microbiology, Faculty of Science, Al-Azhar University, Assiut 71524, Egypt; ^2^Colin Ratledge Center for Microbial Lipids, School of Agricultural Engineering and Food Science, Shandong University of Technology, Zibo 255000, China; ^3^Department of Botany and Microbiology, Faculty of Science, South Valley University, Qena 83523, Egypt

## Abstract

*Pseudomonas aeruginosa* is an organism commonly found in the environment and one of the most common causes of human urinary tract infections in developed and developing countries. The present study aimed to investigate the effect of five medicinal plant extracts on the isolated drug-resistant *P. aeruginosa* clinical isolates. A total of 100 urine samples were collected from Nagaa Hammadi and Qena General Hospitals and private medical analysis laboratories in Qena governorate, Upper Egypt. Samples were screened for the prevalence of UTI pathogens by biochemical tests, antibiotics sensitivity, detection of virulence, and antibiotic-resistant genes by using multiplex PCR. *P. aeruginosa* is by far the subdominant causative agent with a percentage of 14%. Clinical isolates were multidrug-resistant, containing *bla*_TEM_, *bla*_SHV_, *toxA*, *lasB*, *pslA*, and *fliC* resistant and virulence genes. Based on bioactivity, the ethanolic extract of clove (*Syzygium aromaticum*) was the most active extract among tested medicinal plants and had the maximum zone of inhibition sized 23 mm against tested bacteria. The results of the minimum inhibitory concentration (MIC) and the minimum bactericidal concentration (MBC) showed a high decrease of inhibition within a concentration range of (10 to 121.25 mg/mL and 20 to 30 mg/mL, respectively). Further, major compounds of oleic acid (27.22%), guanosine (8.91%), indole (6.83%), 1-eicosene (6.30%), and cis-10-nonadecenoic acid (5.37%) were determined among 12 bioactive compounds in the ethanolic extract of *S. aromaticum* using gas chromatography-mass spectrometry (GC-MS). These results indicated that the ethanolic extract of *S. aromaticum* is a promising antibacterial agent for further studies aiming to control bacterial infections including MDR bacteria and develop novel therapeutic alternatives for the treatment of UTI.

## 1. Introduction

Microbial infections are a common cause of urinary ailments in humans and have become a common cause of nosocomial disorders. A urinary tract infection (UTI) in humans is one of the most predominant illnesses in developing countries representing more than 35% of hospitalized patients [1]. It is common in sexually active females and increases in people with diabetes and patients with sickle cell disease or anatomical malformation of the urinary tract; other factors for UTI are an enlarged prostate gland in men and pregnancy in women raising the risk of infection; also, patients with indwelling bladder catheter are predisposed to bacteriuria and UTIs [2]. The family *Enterobacteriaceae* is the most frequent pathogen detected, causing most of the UTIs, such as *Escherichia coli* (reaching 77%), followed by *Enterococcus faecalis*, *Pseudomonas aeruginosa*, *Klebsiella pneumoniae*, *Acinetobacter* spp., *Proteus* spp., and *Staphylococcus aureus* [3, 4].

Each organism has its encoding virulence gene(s) that is responsible for the pathogenicity of an infective agent [5]. *P. aeruginosa* is well known as an opportunistic bacterial pathogen [6], essentially dangerous to patients suffering from cystic fibrosis and people with a weak immune system [7]. *P. aeruginosa* infection is often difficult to eradicate because of resistance to multiple antibiotics and disinfectants; it can organize itself in a “biofilm” where it can be protected from antibiotics and immune cells, which ranks as the third most common organism after *E. coli* and *Enterococci* associated with urinary tract infections [8]. *P. aeruginosa* clinical isolates are extremely high resistant to multiple antibacterial agents [9] and subsequently have joined the ranks of ‘superbugs' because of its broad capacity to create resistance [10].

Multidrug-resistant (MDR) Gram-negative bacteria, currently endemic in many countries, may cause severe infections that are usually associated with high death rates. MDR is the main concern in developing a definitive therapy and treatments for *P. aeruginosa* infections [11, 12]. Multiple mechanisms of resistance against antibacterial agents have been reported, which include inactivation of drugs by several factors such as enzymatic modification and their actions, target mutation, reduced transport, and/or increased efflux and decreased uptake of the antibiotics [13]. Due to the frequent development of resistance during single-drug therapy treatment of patients associated with *P. aeruginosa*, the natural plant extracts have been reported as an emergent strategy to combat and overcome associated infections and their resistance mechanisms [14, 15].

To improve safer medical drugs, several studies on the antimicrobial activity of plant and herbal extracts have been investigated against drug-resistant organisms. Natural bioactive compounds from medicinal plants are among the alternative sources investigated to replace traditional antibiotics and synthetic antimicrobial agents [14, 16]. The World Health Organization (WHO) estimates that more than 80% of the people worldwide, mainly in developing countries, use multiple plant extracts and their active molecules as folk medicine in conventional drugs [17]. Several studies have been confirmed as significant antibacterial, antifungal, antiviral, and anticarcinogenic activities of some aromatic herbs such as cinnamon (*Cinnamomum zeylanicum*), oregano (*Origanum vulgare*), clove (*Syzygium aromaticum*), thyme (*Thymus vulgaris*), and turmeric (*Curcuma longa*). However, clove has received much attention among other plant spices because it has a great antimicrobial effect against multidrug-resistant and antioxidant activities [18]. In this context, the global emergence of multidrug-resistant bacteria invokes an urgent and imperative necessity for exploring novel antimicrobials against microbial pathogens [19]. Thus, searching for new antibacterial agents, especially from medicinal plants, with narrow-spectrum antibiotics and an actual effective response against resistant pathogenic strains, is an extremely urgent task.

In the present study, we aimed to investigate the antibacterial potential of different medicinal plant extracts against MDR *P. aeruginosa* clinical isolates and their relationship with antimicrobial susceptibility and to determine the resistant patterns of UTI pathogens by multiplex PCR. The chemical constituents of the active plant extract were determined using gas chromatography-mass spectrometry (GC-MS).

## 2. Materials and Methods

### 2.1. Sampling, Data Collection, and Processing

A total of 100 urine samples were collected from the Nagaa Hammadi and Qena General Hospitals and some private medical analysis laboratories in Qena governorate, Upper Egypt, from January 2018 to January 2019. Samples were collected from patients between the ages of 6 and 81 years asymptomatic with high body temperature and chills, inflammation in the urethra, nausea and vomiting, diarrhoea, mucus, and an open wound treated in the hospital, treatments requiring invasive devices like urinary catheters. Clean catch midstream urine specimens were collected into the sterile screw container from each patient who had not received antibiotics within the last 5 days. Each specimen was clearly labelled and transported on dry ice to the microbiology laboratory for further processing. Urine physical condition, guidelines for sample collection, specific gravity, and their pH were determined [20].

### 2.2. Isolation and Identification of UTI Pathogens

Collected urine samples were streaked onto sterile MacConkey agar medium containing peptone 17 g/L; polypeptone 3 g/L; lactose 10 g/L; bile salts 1.5 g/L; sodium chloride 5 g/L; neutral red 3 g/L; crystal violet 0.001 g/L; agar 15 g/L; distilled water 1000 mL and final pH 7.1 ± 0.2 and blood agar medium containing peptone 5 g/L; beef extract 3 g/L; sodium chloride 5 g/L; sheep blood 50 mL; agar 15 g/L; distilled water 1000 mL and final pH 7.4 ± 0.2. Then, plates were incubated at 37°C for 24 h. The obtained pure colonies were subcultured onto nutrient agar (NA) media containing peptone 5 g/L; beef extract 3 g/L; sodium chloride 5 g/L; agar 15 g/L; distilled water 1000 mL and final pH 6.8 ± 0.2 and identified by standard biochemical methods [21].

### 2.3. Antimicrobial Sensitivity Testing

The antibiotic susceptibility testing was performed by the Kirby-Bauer disk diffusion method [22]. Bacterial suspensions used were equal to 0.5 McFarland turbidity (1.5 × 10^8^ CFU/mL). The tryptic soy agar (TSA) (containing pancreatic digest of casein 15 g/L; peptic digest of soybean meal 5 g/L; sodium chloride 5 g/L; agar 15 g/L; distilled water 1000 mL and final pH 7.3 ± 0.2) was seeded with 100 *μ*L of the bacterial suspension according to Clinical and Laboratory Standard Institute [23]. The tested antibacterial agents used in this method were as follows: amikacin (AK 30 *μ*g), gentamicin (CN 10 *μ*g), aztreonam (ATM 30 *μ*g), ceftazidime (CAZ 30 *μ*g), cefoxitin (30 *μ*g), ceftriaxone (30 *μ*g), cefepime (30 *μ*g), ampicillin-sulbactam (20/10 *μ*g), piperacillin-tazobactam (PIT 100/10 *μ*g), imipenem (IPM 10 *μ*g), ciprofloxacin (CIP 5 *μ*g), cefepime (CPM 10 *μ*g), gatifloxacin (GAT 30 *μ*g), norfloxacin (NOR 20 *μ*g), ofloxacin (OF 30 *μ*g), and tobramycin (TOB 10 *μ*g). Interpretation of the results was performed according to Clinical and Laboratory Standards Institute guidelines to determine if the isolate is resistant or susceptible to the tested antibiotics.

### 2.4. Detection of Virulence and Antibiotic-Resistant Genes of Isolated *P. aeruginosa*

Molecular characterization of the recovered urinary tract infection *P. aeruginosa* was carried out by multiplex polymerase chain reaction (PCR). The detected enterotoxins genes for *P. aeruginosa* were *bla*_TEM_, *bla*_SHV_, *bla*_CTX_, *toxA*, *lasB*, *pslA*, and *fliC*; besides, the 16S rDNA gene was performed. The encoding enterotoxins and antibiotic-resistant genes were performed using forward and reverse primers sets. All primer sequences with corresponding references are listed in Table 1.

### 2.5. DNA Amplification for the Selected Virulence and Antibiotic Resistance Genes of Isolates

DNA extraction was carried out according to QIAamp DNA Mini Kit instructions (QIAGEN, Germany, GmbH) as described previously [30] with some modifications. In brief, approximately 200 *μ*L of the sample suspension was inoculated with 10 *μ*L of proteinase K and then with 200 *μ*L of lysis buffer and incubated for 10 min at 56°C. After the incubation period, 200 *μ*L of ethanol (96%) was added to the lysate. The sample was then washed and centrifuged following the manufacturer's instructions. The obtained DNA was eluted with 100 *μ*L of elution buffer provided in the kit. PCR amplification was performed using oligonucleotide primers (Metabion, Germany) that were utilized in a 25 *μ*L reaction containing 12.5 *μ*L of EmeraldAmp GT PCR Master Mix 2x premix (Takara, Japan), 1 *μ*L of each primer, 6 *μ*L PCR grade H_2_O, and 6 *μ*L of DNA template. The Applied Biosystems 2720 Thermal Cycler was used for PCR. Studied primers, amplicon sizes, and cycling conditions are summarized in Table 1. The PCR products after amplification were separated by 1% agarose gel electrophoresis when 20 *μ*L of each product was loaded in each gel slot. To determine the DNA fragment sizes, GeneRuler 100 bp ladder (Fermentas, Thermo), Gelpilot 100 bp, and Gelpilot 100 bp plus ladders (QIAGEN, USA) were used as a marker for electrophoresis. The gel was visualized via a gel documentation system (Alpha Innotech, Biometra) and the data were analyzed using computer software (Automatic Image Capture, USA).

### 2.6. Plant Material

Five medicinal plants used in this study were as follows: turmeric (*Curcuma longa*), clove (*Syzygium aromaticum*), garlic (*Allium sativum*), pond seeds (*Nigella sativa*), and ginger (*Zingiber officinale*); they were collected from commercial sources in Nagaa Hammadi, Qena, Egypt. Before the extraction, the collected plant materials were washed with sterile water and further dried and then ground to obtain a homogenous powder. The plant species were morphologically photographed for documentation and underwent further assisted taxonomic identification at the Department of Botany and Microbiology, Faculty of Science, South Valley University, Qena, Egypt.

### 2.7. Preparation of Plant Extracts

Multiple solvents have been employed including water, absolute ethanol, and ethyl acetate to extract the bioactive compounds from medicinal plants. The dried form of each plant was soaked separately with sterile distilled water, ethanol, and ethyl acetate (100 g in 1 L solvent) for 7 days and extracted by maceration. The obtained extracts were filtered through Buchner funnel with Whatman No.1 filter paper and evaporated by a rotary evaporator (BÜCHI R-114, Switzerland) under reduced pressure to dryness at 45°C. The plant crude extracts were stored at 4°C until use. All extracts were redissolved in dimethyl sulfoxide (DMSO) except the aqueous extract, which redissolved in sterile distilled water at a concentration of 200 mg/mL. Before using bioassay, the reconstituted extract solutions were sterilized by micron syringe filters (0.45 *μ*m).

### 2.8. Antibacterial Screening for the Effectiveness of Selected Plants

The antibacterial activity of the five plants was determined using the standard disc diffusion method as described previously [31]. In brief, Petri plates were prepared with approximately 20 mL of sterile Mueller-Hinton agar (MHA, 17.5 g/L acid hydrolysate of casein, 2.0 g/L beef extract, 1.5 g/L starch, and 18.0 g/L agar in 1000 mL H_2_O, pH 7.3 ± 0.2). Overnight cultures of *P*. *aeruginosa* clinical isolates (5 × 10^4^ spores/mL) were picked up by sterile swab sticks and streaked on the top of the solid media and allowed to dry completely for 20 min. The plant extract stock concentration (100 mg/mL) was prepared by dissolving the extract in diluted dimethyl sulfoxide (10% DMSO) and sterile-filtered through a 0.2 *μ*m pore syringe filter. Sterile Whatman No. 1 filter paper discs of 6 mm diameter were impregnated with each plant crude extract and discs were stored at 4°C before use. Extract-impregnated discs (20 *μ*L) were placed on agar plates and incubated for 24 h at 37°C. Pure 10% DMSO (20 *μ*L) was used as a negative control, while colistin (10 mg/disc) was used as a positive control. Then, antibacterial activity was determined by measuring the diameter of inhibition zones in millimeters (mm) against the test bacterial isolates. The experiments were performed in triplicate and the mean values were noted.

#### 2.8.1. Determination of the Minimum Inhibitory Concentration (MIC) and Minimum Bactericidal Concentration (MBC)

The 2‐(*p*‐iodophenyl)‐3‐(*p*‐nitrophenyl)‐5‐phenyl tetrazolium chloride (INT) reduction assay was used to determine the MIC and MBC as described preciously [32]. Overnight prepared MHB cultures of *P*. *aeruginosa* clinical isolates were adjusted to OD_600_ of 0.5 McFarland, and 100 *μ*L of each bacterial culture was taken into sterilized 96-well microplate. Then, 20 *μ*L of the most active extract was added where ten different concentrations were prepared (10^−1^ to 10^−10^). The 96-well microplate was incubated for 24 h at 37°C. MIC was determined by the addition of 40 *μ*L of (INT) (0.2 mg/mL, Sigma-Aldrich) to the microplate wells and reincubated for 30 min at 37°C; colistin (20%) was used as a positive control. MIC was defined as the lowest concentration at which colour changes [33]. MBC was determined as previously described [34].

### 2.9. GC-MS Determination of Bioactive Compounds

The selected active solvent extract was dissolved in methanol (100%) and dehydrated with anhydrous sodium sulphate and then filtered through a syringe filter (0.45 *μ*m pore size) before injection. A Trace GC1310-ISQ mass spectrometer (Thermo Scientific, Austin, TX, USA) was used for the chromatographic analysis, and the compounds were separated with a direct capillary column TG–5MS (30 m × 0.25 mm × 0.25 *μ*m film thickness). The column temperature was initially 50°C and then increased by 5°C/min to 230°C with holding 2 min and then increased to 290°C at 10°C/min. The injector and MS transfer line temperatures were kept at 250 and 260°C, respectively. Helium was used as a carrier gas at a stable flow rate of 1 mL/min. The solvent delay was 3 min, and a diluted sample of 1 *μ*L was injected automatically using an autosampler AS1300 coupled with GC in the split mode. EI mass spectra were generated at an ionisation voltage of 70 eV with a mass scan of 40–1000 amu. The ion source temperature was set at 200°C. The extract components were identified by comparison of their retention times and mass spectra with those of WILEY 09 and NIST 11 mass spectral database.

### 2.10. Statistical Analysis

All the experiments were performed in triplicate. Data were presented as mean ± SD. GraphPad Prism 6.0 (GraphPad Software, San Diego, CA, USA, https://www.graphpad.com) was used to calculate one-way analysis of the variance (ANOVA) with multiple comparison tests (Tukey's) to evaluate the effect of the different extract.

## 3. Results

### 3.1. Prevalence of UTI Associated Bacteria

**Figure 1:**
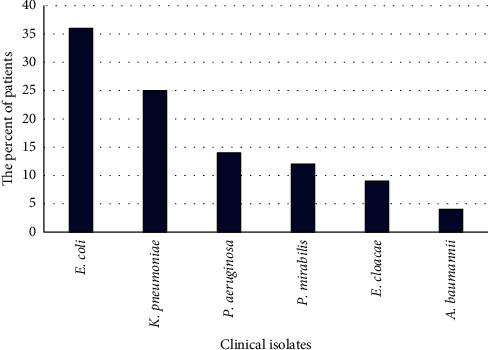
Prevalence of different clinical isolates in urine samples.

The incidence of the isolated urinary tract infection pathogens in urine sample was recorded from 35% males and 65% females patients (35 males and 65 females), represented by age under 30 years (10.77 and 14.29%), from 30 to 60 years (81.54 and 68.57%), and more than 60 years (7.69 and 17.14%) for females and males, respectively. All the 100 urine samples that have been properly collected were positive for bacterial growth. The most common predominant organism was *E. coli* with a percentage of 36%, followed by *K. pneumoniae* 25%, *P. aeruginosa* 14%, *Proteus mirabilis* 12%, *Enterobacter cloacae* 9%, and *Acinetobacter baumannii* 4% (Figure 1). Total aerobic bacterial count in those samples ranged from 1.9 to 2.10 × 10^6^ CFU/mL, and *P. aeruginosa* isolates (P1 to P14) were preliminary identified by biochemical characterizations and selected for further study (see Table S1 in the Supplementary Materials).

### 3.2. Antibiotics Sensitivity of *P. aeruginosa* Clinical Samples

The resistance rate of all isolated UTIs *P. aeruginosa* clinical samples against multiple antibiotics with different influence is shown in Table 2. The results showed that the rate of resistance to all tested antibiotics was 100% in *P. aeruginosa* isolates (P1, P2, P3, P4, P5, P6, P7, P9, P10, and P11). While the resistance level to all antibiotics was noticed in all isolated samples except *P. aeruginosa* (P4 and P6), they were sensitive for piperacillin-tazobactam. Interestingly, the susceptibility levels of aztreonam, cefepime, ciprofloxacin, norfloxacin, ofloxacin, and piperacillin-tazobactam were observed only against *P. aeruginosa* isolates (P12, P13, and P14), followed by *P. aeruginosa* (P8) that was resistant to 78.5% of all tested antibiotics. Finally, the sensitivity level of imipenem was detected only against *P. aeruginosa* isolates (P8 and P14).

### 3.3. Detection of Antibiotic Resistance and Virulence Genes

**Figure 2:**
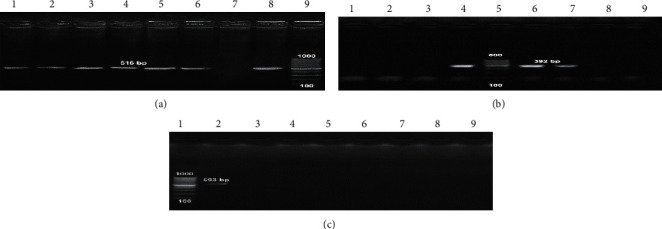
Agarose gel electrophoresis of multiplex PCR of positive *P. aeruginosa* isolates (6 isolates) carrying antibiotic-resistant genes. (a) *bla*_TEM_ antibiotic-resistant gene; lanes 1–6: *P. aeruginosa* isolates P14, P13, P12, P9, P8, and P4 at 516 bp, lane 7: negative control for detected genes; lane 8: positive control of DNA confirmed by reference laboratory for quality control; lane 9: 100 bp ladder as molecular size DNA marker (cat. no. 239035) supplied from QIAGEN (USA). (b) *bla*_SHV_ antibiotic-resistant gene; lanes 6 and 7: only 2 *P. aeruginosa* P8 and P9 were *bla*_SHV_ positive; lane 4: positive control of DNA confirmed by reference laboratory for quality control; lane 5: 100 bp ladder as molecular size DNA marker (cat. no. 239035) supplied from QIAGEN (USA). Other lanes were negative *bla*_SHV_ results. (c) *bla*_CTX_ antibiotic-resistant gene; all *P. aeruginosa* isolates were *bla*_CTX_ negative; lane 1: 100 bp ladder as molecular size DNA marker (cat. no. 239035) supplied from QIAGEN (USA) lane 2: positive control of DNA.

**Figure 3:**
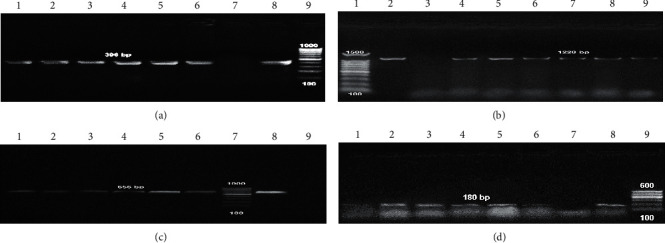
Agarose gel electrophoresis of multiplex PCR of virulence genes characterized for *P. aeruginosa* isolates (6 isolates). Virulence-associated genes were detected in all *P. aeruginosa* isolates at 396, 1220, 656, and 180 bp in *toxA*, *lasB*, *pslA*, and *fliC*, respectively. (a) *toxA* and (d) *fliC* virulence genes; lane 7: negative control for detected genes; lane 8: positive control of DNA confirmed by reference laboratory for quality control; lane 9: gel pilot 100 bp ladder as molecular size DNA marker (cat. no. 239045) supplied from QIAGEN (USA). (b) *lasB* virulence gene; lane 3: negative control for detected genes; lane 2: positive control of DNA confirmed by reference laboratory for quality control; lane 1: gel pilot 100 bp ladder as molecular size DNA marker (cat. no. 239045) supplied from QIAGEN (USA). (c) *pslA* virulence gene; lane 9: negative control for detected genes; lane 8: positive control of DNA confirmed by reference laboratory for quality control; lane 7: gel pilot 100 bp ladder as molecular size DNA marker (cat. no. 239045) supplied from QIAGEN (USA).

To detect the antibiotic-resistant genes in addition to virulence determinants among *P. aeruginosa* isolates, the multiplex PCR screening was performed. Three different genes (*bla*_TEM_, *bla*_SHV_, and *bla*_CTX_) were applied in this experiment, and the results showed that antibiotic-resistant gene *bla*_TEM_ was present in 6/14 *P. aeruginosa* isolates (42.8%). *bla*_SHV_ gene was positive among only two isolates P8 and P9 (16.3%). Finally, all tested clinical isolates do not contain *bla*_CTX_ antibiotic-resistant gene (Table 2; Figure 2). On the other hand, the multiple virulence genes *toxA*, *lasB*, *pslA*, and *fliC* were present in tested *P. aeruginosa* isolates (P4, P8, P9, P12, P13, and P14). However, these virulence genes were not detected in other isolates (Table 2; Figure 3).

### 3.4. Antibacterial Activity Screening for Selected Plant Extracts

The antibacterial activity of the five plant species was investigated against selected MDR *P. aeruginosa* using the disc diffusion method (Table 3). The results revealed that two plant extracts were potentially effective in suppressing *P. aeruginosa* growth with variable potency. The ethyl acetate and ethanolic extracts of *C. longa* showed varying degrees of antibacterial activities against tested clinical isolates ranging from 7 to 18 mm, while the aqueous, ethanolic, and ethyl acetate extracts of *S. aromaticum* exhibited high percentages of inhibition ranging 7–12, 6–23, and 12–15 mm, respectively, against tested bacteria. On the other hand, the antibacterial activity of *Z. officinale*, *N. sativa*, and *A. sativum* showed negative effects and all the tested bacteria were resistant to these three plant extracts. Results of antimicrobial activity of the five plant extracts suggested that the ethanolic extract of *S. aromaticum* was the most effective among other extracts and showed strong antibacterial activity against selected MDR strains. Hence, experiments were conducted to determine their minimal inhibitory concentration (MIC) and minimal bactericidal concentration (MBC) against *P. aeruginosa* bacterial strains. Moreover, chemical analysis of *S. aromaticum* active extract was performed.

### 3.5. Minimum Inhibitory and Bacteriostatic Concentration

**Figure 4:**
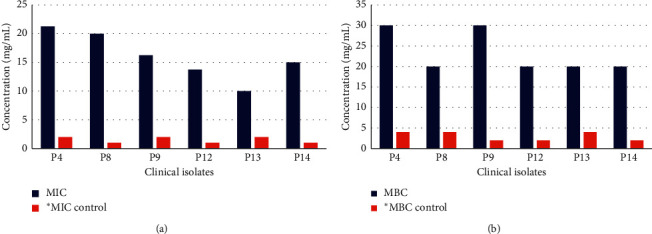
MIC (a) and MBC (b) values of *S. aromaticum* ethanolic extract (100 mg/mL) against selected *P. aeruginosa*. ^*∗*^Colstin as control (10 mg/mL).

The MIC and MBC were the lowest and bactericidal concentrations that inhibited bacterial growth, respectively, and were determined using the microdilution method with the help of (INT) reduction assay. MIC values of *S. aromaticum* ethanolic extract ranged from 10 to 21.25 mg/mL, while the MIC of colistin (positive control) was 1 to 2 mg/mL against all the tested isolates. The ethanolic extracts from *S. aromaticum* displayed the minimum MIC activity against bacterial isolate P13 with a MIC value of 10 mg/mL among tested isolates, while P14 showed the maximum MIC value at 21.25 mg/mL. As for MBC, the ethanolic extract of *S. aromaticum* showed high bactericidal activity at a concentration of 20 to 30 mg/mL. A bactericidal action was observed at a concentration of 30 mg/mL in the case of P4 and P9 isolates, while in the case of other tested isolates, MBC is 20 mg/mL. MBC of colistin (positive control) ranged from 2 to 4 mg/mL (Figure 4).

### 3.6. Identification of Bioactive Compounds by GC-MS

**Figure 5:**
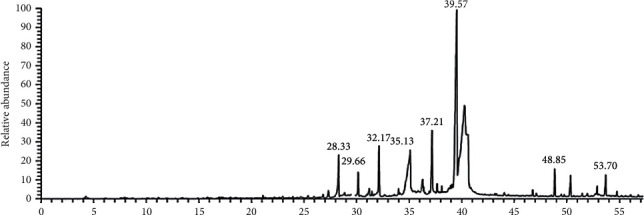
A typical chromatogram of the biologically active compounds present in the ethanolic extract of *S. aromaticum*.

The bioactive chemical compounds present in the ethanolic extract obtained from *S. aromaticum* are analyzed by GC-MS (Table 4). The GC-MS analysis data showed the presence of 12 known bioactive compounds in the plant ethanolic extract, which were likely responsible for the antibacterial activity found in the plant extract against the tested bacteria. The retention time, molecular formula, and peak area of these compounds were also presented. Based on abundance, the highest five major compounds present in the ethanolic extract were oleic acid (27.22%), guanosine (8.91), indole (6.83%), 1-eicosene (6.30%), and cis-10-nonadecenoic acid (5.37%), and their detected compounds peak are ranged from 0.36 to 2.54%. The GC chromatogram with their concentration peak area (%), peak number, and retention time (RT) is presented (Table 4; Figure 5).

## 4. Discussion

During the past few decades, the prevalence of microbial infections has increased significantly. Continuous use of antimicrobial drugs in treating such infections has led to the emergence of resistance among the various microbial strains. MDR is defined as a simultaneous resistance of an organism to the administered antimicrobial drugs that are structurally unrelated and have different molecular targets, despite its sensitivity [35, 36]. Many different types of MDR bacteria such as methicillin-resistant *Staphylococcus aureus* (MRSA), vancomycin-resistant *Enterococcus* (VRE), penicillin-resistant *Streptococcus pneumoniae*, *P. aeruginosa*, and *E. coli* have been paid increased attention as their potential bacterial pathogenicity and human infections [37]. Drug resistance of mainly *P. aeruginosa* from different clinical specimens in Egypt is one of the main active pathogens in Egyptians, and these organisms associated with UTIs have been little studied [38].

In the present study, 100 urine samples were collected from asymptomatic UTIs patients in Nagaa Hammadi and Qena General Hospitals and other medical laboratories; all collected samples had UTIs with predominant organisms such as *E. coli*, *K. pneumoniae, P. aeruginosa*, *P. mirabilis*, *E. cloacae*, and *A. baumannii*. Moreover, *P. aeruginosa* isolated strains were selected for the current study and the emergence of antibiotic resistance of *P. aeruginosa* is a worldwide threat as it affects persons globally. According to our analysis results of the urinary samples, approximately 71.5% (*n* = 10/14) of *P. aeruginosa* isolates were resistant to tested antibiotics. A high rate of antibiotic resistance was also demonstrated in a similar Egyptian research study in which 45% of *P. aeruginosa* isolates exhibited resistance to ceftazidime antibiotics [39]. Also, other studies showed a 91% resistance rate in other regions of Egypt [40]. Prior studies by Rodríguez-Martínez [41] showed that approximately 87% of *P. aeruginosa* isolates were resistant against imipenem, and 100% resistance against ciprofloxacin was reported by Tam et al. [42], which is in agreement with our findings. Our study was also supported by Meenakumari et al., where amikacin and gentamicin antibiotics were resistant to *P. aeruginosa* clinical isolates [43]. Contrary to our study, *P. aeruginosa* strains showed 20 and 100% resistance to cotrimoxazole, which were reported in Libya and Nigeria, respectively [44, 45].

Our data showed that *bla*_TEM_ antibiotic-resistant gene was present in *P. aeruginosa* isolates (42.8%), while *bla*_SHV_ was positive only in two isolates (16.3%). Interestingly, all tested isolates do not contain *bla*_CTX_ antibiotic-resistant gene. On the other hand, the virulence genes *toxA*, *lasB*, *pslA*, and *fliC* were detected in *P. aeruginosa* isolates (42.8%). A study in 2018 by Al Dawodeyah et al. [46] reported that the positive results for *bla*_CTX-M_, *bla*_TEM_, *bla*_VEB_, *bla*_SHV_, and *bla*_GES_ genes were detected in *P. aeruginosa* clinical isolates with the percentage of 68.9, 18.9, 18.9, 12.5, and 15.6%, respectively. They found no specific relation between the resistance to antibiotics, the existence of virulence genes, and their genotypes between MDR *P. aeruginosa* isolates.

Bioactive products from medicinal plants have been studied for decades, which is one of the most successful sources of drugs to treat bacterial infections. Accordingly, there is urgent demand for the discovery of novel antibiotics with new modes of action against multidrug-resistant bacteria. Antimicrobial constituents of natural origin have the potential to play a great therapeutic role in treating infectious diseases [47]. Antibacterial activity of five plants and their sequential extracts (aqueous, ethanol, and ethyl acetate) were subjected to bioactivity screening against six MDR *P. aeruginosa* bacterial strains that exhibited resistance to various antibiotics. Our results showed that only ethanolic extracts of *S. aromaticum* and *C. longa* were highly effective against *P. aeruginosa* isolates with maximum inhibition zone ranging between 23 and 10 mm, respectively, while other tested plants and their extracts had no activity against tested bacteria (100% resistant). Similar results reported by Alharbi [48] confirmed that the ethanolic extract of *S. aromaticum* has bactericidal activity on *P. aeruginosa* ATCC 27853 and bacteriostatic on *P. aeruginosa* clinical isolates. Other research works conducted in parallel investigated the highly effective methanolic extract of *S. aromaticum* against MDR *P. aeruginosa* [49]. In the earlier study [50], they focused on the activity of medicinal plants such as *Aloe vera* and *C. longa,* which showed antimicrobial activity against Gram-negative bacteria. Our study can be supported by the work of Aziz et al. [51], who demonstrated that the extracts of *S. aromaticum* had remarkable antimicrobial activities and showed a convincing zone of inhibition against the tested MDR *P. aeruginosa* strains, and they are relatively more susceptible than the other Gram-negative, especially those isolated from urine samples of UTI patients. These results can be satisfied by the data of previous studies in which the *S. aromaticum* ethanolic extracts showed the maximum inhibition potential towards MDR bacteria [52].

The bioactive constituents responsible for its activity of *S. aromaticum* have been determined by GC analysis. The GC-MS analysis profile of the plant extract revealed 12 major compounds. Oleic acid, guanosine, indole, 1-eicosene, and cis-10-nonadecenoic acid were the main compounds in the ethanolic extract of *S. aromaticum*. A previous study showed that the oleic acid produced by marine bacteria inhibits pathogenic Gram-negative bacteria and might indicate an influence in the medical treatments [53–55]. Other important active compounds such as hexacosanol, indole, and dodecanoic acid have been reported with a wide range of pharmaceutical activities, such as antibacterial, anticancer, and antitumor activities [53–55].

## 5. Conclusions

The analysis of the activity of biomolecules produced by medicinal plants, particularly *S. aromaticum*, is important due to the decreased efficiency of known antimicrobials, and it is necessary to explore new therapeutic alternatives for the treatment of UTIs. To the best of our knowledge, this study may be the first in Upper Egypt to investigate the potential activity of *S. aromaticum* ethanolic against the multidrug-resistant *P. aeruginosa* clinical strains, which carry antibiotic resistance and virulence genes as confirmed by multiplex PCR. However, the isolation of compounds from the *S. aromaticum* is a great challenge that should be considered to use these individual compounds for biomedical therapeutics against MDR bacteria. This research may serve as a fruitful platform to explore and test the obtained compounds against uropathogens.

## Figures and Tables

**Table 1:** Target genes, primers, sequences, cycling conditions, and amplicon sizes. All specific sequences were amplified in this experiment (Metabion, Germany). A 16Sr DNA gene of *P. aeruginosa* was also examined.

Target gene	Sequence	Amplified product (bp)	Primary denaturation	Amplifications (35 cycles)				References
				Secondary denaturation	Annealing	Extension	Final extension	
*P. aeruginosa* 16Sr DNA	GGGGGATCTTCGGACCTCA TCCTTAGAGTGCCCACCCG	956	94°C/5 min	94°C/30 sec	52°C/45 sec	72°C/1 min	72°C/10 min	[24]

*Antibiotic-resistant genes*								
*bla*_TEM_	ATCAGCAATAAACCAGC CCCCGAAGAACGTTTTC	516	94°C/5 min	94°C/30 sec	54°C/45 sec	72°C/45 sec	72°C/10 min	[25]
*bla*_SHV_	AGGATTGACTGCCTTTTTG ATTTGCTGATTTCGCTCG	392	94°C/5 min	94°C/30 sec	54°C/45 sec	72°C/45 sec	72°C/10 min	[25]
*bla*_CTX_	ATGTGCAGYACCAGTAARTKATGGC TGGGTRAARTARGTSACCAGAAYCAGCGG	593	94°C/5 min	94°C/30 sec	54°C/40 sec	72°C/45 sec	72°C/10 min	[26]

*Virulence genes*								
*toxA*	GACAACGCCCTCAGCATCACCAGC CGCTGGCCCATTCGCTCCAGCGCT	396	94°C/5 min	94°C/30 sec	55°C/40 sec	72°C/40 sec	72°C/10 min	[27]
*lasB*	ACAGGTAGAACGCACGGTTG GATCGACGTGTCCAAACTCC	1220	94°C/5 min	94°C/30 sec	54°C/40 sec	72°C/1 min	72°C/12 min	[28]
*pslA*	TCCCTACCTCAGCAGCAAGC TGTTGTAGCCGTAGCGTTTCTG	656	94°C/5 min	94°C/30 sec	60°C/40 sec	72°C/45 sec	72°C/10 min	[29]
*fliC*	TGAACGTGGCTACCAAGAACG TCTGCAGTTGCTTCACTTCGC	180	94°C/5 min	94°C/30 sec	56.2°C/30 sec	72°C/30 sec	72°C/7 min	[29]

**Table 2:** Antimicrobial sensitivity, antibiotic-resistant genes, and virulence genes of multidrug-resistant *P. aeruginosa* from urine samples.

Clinical isolates	^*∗*^P1	P2	P3	P4	P5	P6	P7	P8	P9	P10	P11	P12	P13	P14
*Antimicrobial agents (μg)* ^*∗∗*^														
AK	R	R	R	R	R	R	R	R	R	R	R	R	R	R
ATM	R	R	R	R	R	R	R	R	R	R	R	S	S	S
CPM	R	R	R	R	R	R	R	R	R	R	R	S	S	S
CAZ	R	R	R	R	R	R	R	R	R	R	R	R	R	R
CIP	R	R	R	R	R	R	R	R	R	R	R	S	S	S
GAT	R	R	R	R	R	R	R	R	R	R	R	R	R	R
CN	R	R	R	R	R	R	R	R	R	R	R	R	R	R
IPM	R	R	R	R	R	R	R	S	R	R	R	R	R	S
NOR (urine)	R	R	R	R	R	R	R	S	R	R	R	S	S	S
OF	R	R	R	R	R	R	R	R	R	R	R	S	S	S
PIT	R	R	R	S	R	S	R	R	R	R	R	S	S	S
TOB	R	R	R	R	R	R	R	S	R	R	R	R	R	R

*Antibiotic-resistant genes* ^*∗∗∗*^														
*bla*_TEM_	ND	ND	ND	+	ND	ND	ND	+	+	ND	ND	+	+	+
*bla*_SHV_	ND	ND	ND	−	ND	ND	ND	+	+	ND	ND	−	−	−
*bla*_CTX_	ND	ND	ND	−	ND	ND	ND	−	−	ND	ND	−	−	−

*Virulence genes* ^*∗∗∗∗*^														
*toxA*	ND	ND	ND	+	ND	ND	ND	+	+	+	ND	+	+	+
*lasB*	ND	ND	ND	+	ND	ND	ND	+	+	+	ND	+	+	+
*pslA*	ND	ND	ND	+	ND	ND	ND	+	+	+	ND	+	+	+
*fliC*	ND	ND	ND	+	ND	ND	ND	+	+	+	ND	+	+	+

^*∗*^14 *P. aeruginosa* different strains (P1–P14). ^*∗∗*^Antibiotics: AK: amikacin; ATM: aztreonam; CPM: cefepime; CAZ: ceftazidime; CIP: ciprofloxacin; GAT: gatifloxacin; CN: gentamicin; IPM: imipenem; NOR: norfloxacin; OF: ofloxacin; PIT: piperacillin-tazobactam; TOB: tobramycin. R = resistant; S = sensitive. ^*∗∗∗*^Antibiotic-resistant genes, ^*∗∗∗∗*^virulence genes; +: present; –: absent; ND, not determined.

**Table 3:** Antibacterial screening of tested plant extracts (100 mg/mL) against the six clinical isolates of *P. aeruginosa*.

Tested plants	Inhibition effect diameter (mm)														
	*S. aromaticum*			*C. longa*			*A. sativum*			*N. sativa*			*Z. officinale*		
Clinical isolates	H_2_O extract	EtOH extract	EtOAc extract	H_2_O extract	EtOH extract	EtOAc extract	H_2_O extract	EtOH extract	EtOAc extract	H_2_O extract	EtOH extract	EtOAc extract	H_2_O extract	EtOH extract	EtOAc extract
P4	7 ± 0.2	16 ± 0.1	14 ± 0.1	18 ± 0.2	9 ± 0.1	8 ± 0.1	—	—	—	—	—	—	—	—	—
Positive control*∗*		21 ± 0.36			20 ± 0.21			19 ± 1.6			14 ± 0.19			19 ± 0.04	
P8	—	6 ± 0.2	15 ± 0.3	13 ± 0.6	10 ± 0.1	8 ± 0.2	—	—	—	—	—	—	—	—	—
Positive control		15 ± 0.31			15 ± 0.29			14 ± 0.11			15 ± 0.27			15 ± 0.14	
P9	8 ± 0.5	23 ± 1.6	15 ± 0.8	12 ± 0.2	10 ± 0.1	7 ± 0.1	—	—	—	—	—	—	—	—	—
Positive control		23 ± 1.3			14 ± 0.8			11 ± 0.1			12 ± 0.3			9 ± 0.01	
P12	12 ± 0.1	15 ± 0.3	13 ± 0.2	11 ± 0.1	8 ± 0.2	7 ± 0.1	—	—	—	—	—	—	—	—	—
Positive control		16 ± 1.1			12 ± 0.6			14 ± 0.26			9 ± 0.1			7 ± 0.06	
P13	11 ± 0.3	17 ± 0.5	12 ± 0.1	10 ± 0.1	8 ± 0.1	8 ± 0.2	—	—	—	—	—	—	—	—	—
Positive control		18 ± 0.25			13 ± 0.36			15 ± 0.27			10 ± 0.14			8 ± 0.1	
P14	10 ± 0.1	20 ± 0.6	13 ± 0.2	11 ± 0.1	9 ± 0.1	8 ± 0.1	—	—	—	—	—	—	—	—	—
Positive control		15 ± 1.3			13 ± 0.1			16 ± 0.6			14 ± 0.1			14 ± 0.3	

^*∗*^Colistin (10 mg/disc); P4–P14: selected strains; —, no activity; inhibition zones including the diameter of the paper disc (6 mm). Data are means of three replicates (*n* = 3) ± standard error. It was confirmed that 10% DMSO had no inhibitory effect on any isolate.

**Table 4:** Bioactive chemical components of the ethanolic extract from *S. aromaticum*.

No.	Compounds	Chemical formula	Molecular weight	RT (min)	Area (%)
1	Pentenenitrile	C_5_H_7_N	81	28.33	4.37
2	Ethyl oleate	C_20_H_38_O_2_	310	29.66	2.54
3	cis-10-Nonadecenoic acid	C_19_H_36_O_2_	296	32.17	5.37
4	Indole	C_8_H_7_N	117	35.12	6.83
5	Guanosine	C_10_H_13_N_5_O_5_	283	37.21	8.91
6	Oleic acid	C_18_H_34_O_2_	282	39.55	27.22
7	Chlorozotocin	C_9_H_16_ClN_3_O_7_	313	39.75	0.52
8	1-Eicosene	C_20_H_40_	280	40.62	6.30
9	Nonadecene	C_19_H_38_	266	46.77	1.20
10	3-Hexacosanol	C_26_H_54_O	382	48.85	2.21
11	Nonacosane	C_29_H_60_	408	51.96	0.36
12	Dodecanoic acid	C_12_H_24_O_2_	200	53.70	2.34

## Data Availability

No data were used to support this study.

## Supplementary Materials

Supplementary Materials Table S1: biochemical identification of *P. aeruginosa* isolates by Vitek 2 system.
