# Supplementary material for: Efficacy of Ethanolic Extract of Syzygium aromaticum in the Treatment of Multidrug-Resistant Pseudomonas aeruginosa Clinical Isolates Associated with Urinary Tract Infections
Source: Evid Based Complement Alternat Med. 2021 Jun 15;2021:6612058. doi: 10.1155/2021/6612058 (PMC8221855; doi:10.1155/2021/6612058)
Supplement: Supplementary Materials — Table S1: biochemical identification of P. aeruginosa isolates by Vitek 2 system. [file 6612058.f1.docx]

**Table S1.** Biochemical identification of *P. aeruginosa* isolates by Vitek 2 system

| P14 | P13 | P12 | P11 | P10 | P9 | P8 | P7 | P6 | P5 | P4 | P3 | P2 | P1 |  |
| --- | --- | --- | --- | --- | --- | --- | --- | --- | --- | --- | --- | --- | --- | --- |
| ‒ | ‒ | ‒ | ‒ | ‒ | ‒ | ‒ | ‒ | ‒ | ‒ | ‒ | ‒ | ‒ | ‒ | Ala-Phe-Pro-ARYLAMIDASE |
| ‒ | ‒ | ‒ | ‒ | ‒ | ‒ | ‒ | ‒ | ‒ | ‒ | ‒ | ‒ | ‒ | ‒ | ADONITOL |
| ‒ | ‒ | ‒ | ‒ | ‒ | ‒ | ‒ | ‒ | ‒ | ‒ | ‒ | ‒ | ‒ | ‒ | L-Pyrrolydonyl- ARYLAMIDASE |
| ‒ | ‒ | ‒ | ‒ | ‒ | ‒ | ‒ | ‒ | ‒ | ‒ | ‒ | ‒ | ‒ | ‒ | L-ARABITOL |
| ‒ | ‒ | ‒ | ‒ | ‒ | ‒ | ‒ | ‒ | ‒ | ‒ | ‒ | ‒ | ‒ | ‒ | D-CELLOBIOSE |
| ‒ | ‒ | ‒ | ‒ | ‒ | ‒ | ‒ | ‒ | ‒ | ‒ | ‒ | ‒ | ‒ | ‒ | BETA-GALACTOSIDASE |
| ‒ | ‒ | ‒ | ‒ | ‒ | ‒ | ‒ | ‒ | ‒ | ‒ | ‒ | ‒ | ‒ | ‒ | H_2_S PRODUCTION |
| ‒ | ‒ | ‒ | ‒ | ‒ | ‒ | ‒ | ‒ | ‒ | ‒ | ‒ | ‒ | ‒ | ‒ | BETA-N-ACETYL-GLUCOSAMINIDASE |
| ‒ | ‒ | + | ‒ | + | ‒ | + | ‒ | + | ‒ | ‒ | ‒ | + | ‒ | Glutamyl Arylamidase pNA |
| + | + | + | + | + | + | + | + | + | + | + | + | + | + | D-GLUCOSE |
| + | + | + | + | + | + | + | + | + | + | + | + | + | + | GAMMA-GLUTAMYL-TRANSFERASE |
| ‒ | ‒ | ‒ | ‒ | ‒ | ‒ | ‒ | ‒ | ‒ | ‒ | ‒ | ‒ | ‒ | ‒ | FERMENTATION/ GLUCOSE |
| ‒ | ‒ | ‒ | ‒ | ‒ | ‒ | ‒ | ‒ | ‒ | ‒ | ‒ | ‒ | ‒ | ‒ | BETA-GLUCOSIDASE |
| + | + | ‒ | + | ‒ | + | ‒ | + | ‒ | + | + | + | ‒ | + | D-MALTOSE |
| ‒ | ‒ | ‒ | ‒ | ‒ | ‒ | ‒ | ‒ | ‒ | ‒ | ‒ | ‒ | ‒ | ‒ | D-MANNITOL |
| ‒ | ‒ | + | ‒ | + | ‒ | + | ‒ | + | ‒ | ‒ | ‒ | + | ‒ | D-MANNOSE |
| ‒ | ‒ | ‒ | ‒ | ‒ | ‒ | ‒ | ‒ | ‒ | ‒ | ‒ | ‒ | ‒ | ‒ | BETA-XYLOSIDASE |
| + | + | + | + | + | + | + | + | + | + | + | + | + | + | BETA-Alanine-arylamidase pNA |
| + | + | + | + | + | + | + | + | + | + | + | + | + | + | L-P roline ARYLAMIDASE |
| ‒ | ‒ | + | ‒ | + | ‒ | + | ‒ | + | ‒ | ‒ | ‒ | + | ‒ | LIPASE |
| ‒ | ‒ | ‒ | ‒ | ‒ | ‒ | ‒ | ‒ | ‒ | ‒ | ‒ | ‒ | ‒ | ‒ | PALATINOSE |
| + | + | - | + | - | + | - | + | - | + | + | + | - | + | Tyrosine ARYLAMIDASE |
| ‒ | ‒ | ‒ | ‒ | ‒ | ‒ | ‒ | ‒ | ‒ | ‒ | ‒ | ‒ | ‒ | ‒ | UREASE |
| ‒ | ‒ | ‒ | ‒ | ‒ | ‒ | ‒ | ‒ | ‒ | ‒ | ‒ | ‒ | ‒ | ‒ | D-SORBITOL |
| ‒ | ‒ | ‒ | ‒ | ‒ | ‒ | ‒ | ‒ | ‒ | ‒ | ‒ | ‒ | ‒ | ‒ | SACCHAROSE/SUCROSE |
| ‒ | ‒ | ‒ | ‒ | ‒ | ‒ | ‒ | ‒ | ‒ | ‒ | ‒ | ‒ | ‒ | ‒ | D-TAGATOSE |
| ‒ | ‒ | ‒ | ‒ | ‒ | ‒ | ‒ | ‒ | ‒ | ‒ | ‒ | ‒ | ‒ | ‒ | D-TREHALOSE |
| + | + | + | + | + | + | + | + | + | + | + | + | + | + | CITRATE (SODIUM) |
| + | + | + | + | + | + | + | + | + | + | + | + | + | + | MALONATE |
| ‒ | ‒ | ‒ | ‒ | ‒ | ‒ | ‒ | ‒ | ‒ | ‒ | ‒ | ‒ | ‒ | ‒ | 5-KETO-D-GLUCONATE |
| + | + | + | + | + | + | + | + | + | + | + | + | + | + | L-LACTATE alkalinisation |
| ‒ | ‒ | ‒ | ‒ | ‒ | ‒ | ‒ | ‒ | ‒ | ‒ | ‒ | ‒ | ‒ | ‒ | ALPHA-GLUCOSIDASE |
| + | + | + | + | + | + | + | + | + | + | + | + | + | + | SUCCINATE alkalinisation |
| ‒ | ‒ | ‒ | ‒ | ‒ | ‒ | ‒ | ‒ | ‒ | ‒ | ‒ | ‒ | ‒ | ‒ | BETA-N-ACETYL-GALACTOSAMINIDASE |
| ‒ | ‒ | ‒ | ‒ | ‒ | ‒ | ‒ | ‒ | ‒ | ‒ | ‒ | ‒ | ‒ | ‒ | ALPHA-GALACTOSIDASE |
| ‒ | ‒ | ‒ | ‒ | ‒ | ‒ | ‒ | ‒ | ‒ | ‒ | ‒ | ‒ | ‒ | ‒ | PHOSPHATASE |
| ‒ | ‒ | ‒ | ‒ | ‒ | ‒ | ‒ | ‒ | ‒ | ‒ | ‒ | ‒ | ‒ | ‒ | Glcine ARYLAMIDASE |
| ‒ | ‒ | ‒ | ‒ | ‒ | ‒ | ‒ | ‒ | ‒ | ‒ | ‒ | ‒ | ‒ | ‒ | ORNITHINE DECARBOXYLASE |
| ‒ | ‒ | ‒ | ‒ | ‒ | ‒ | ‒ | ‒ | ‒ | ‒ | ‒ | ‒ | ‒ | ‒ | LYSINE DECARBOXYLASE |
| + | + | ‒ | + | ‒ | + | ‒ | + | ‒ | + | + | + | ‒ | + | DECARBOXYLASE BASE |
| + | + | ‒ | + | ‒ | + | ‒ | + | ‒ | + | + | + | ‒ | + | L- HISTIDINE assimilation |
| ‒ | ‒ | ‒ | ‒ | ‒ | ‒ | ‒ | ‒ | ‒ | ‒ | ‒ | ‒ | ‒ | ‒ | COUMARATE |
| + | + | ‒ | + | ‒ | + | ‒ | + | ‒ | + | + | + | ‒ | + | BETA-GLUCORONIDASE |
| ‒ | ‒ | ‒ | ‒ | ‒ | ‒ | ‒ | ‒ | ‒ | ‒ | ‒ | ‒ | ‒ | ‒ | O/129 RESISTANCE (comp.vibrio) |
| + | + | ‒ | + | ‒ | + | ‒ | + | ‒ | + | + | + | ‒ | + | GLU-GLY-Arg- ARYLAMIDASE |
| ‒ | ‒ | ‒ | ‒ | ‒ | ‒ | ‒ | ‒ | ‒ | ‒ | ‒ | ‒ | ‒ | ‒ | L-MALATE assimilation |
| + | + | ‒ | + | ‒ | + | ‒ | + | ‒ | + | + | + | ‒ | + | ELLMAN |

¬; negative results, +; positive results
